# Subjective expectation of reaching age 85: agreement with population statistics and association with behavioral and psychological factors

**DOI:** 10.1186/s12877-023-03951-w

**Published:** 2023-04-20

**Authors:** Subash Thapa, Jesper B. Nielsen

**Affiliations:** Research Unit of General Practice, University of Southern Denmark, J.B. Winsløws Vej 9, 5000 Odense, Denmark

**Keywords:** Biopsychosocial framework, Decade of healthy aging, Healthy aging, Health literacy, Internal locus of control, Optimism, Life expectancy, Pensions and saving, Subjective life expectation

## Abstract

**Background:**

It is not clearly known how well Danes estimate their chances of reaching the average life expectancy and whether identifiable population subgroups misestimate their life expectancy, and potentially also investments and savings in health and pensions. Therefore, in this study, we examined on the individual level whether subjective life expectancy is in line with the statistically calculated chance of reaching age 85, and further explored the psychological and behavioral factors associated with under or overestimation.

**Methods:**

We opted for a cross-sectional survey design based on a sample of 5,379 Danish citizens aged 50–70 years, returning a web-based questionnaire with socio-demographic data supplemented from a national registry. Average participant estimates of their chance of reaching age 85 for each age range and sex group were compared with actuarial data. We then performed multiple linear regression analyses to examine factors associated with the subjective expectancy of reaching age 85 years.

**Results:**

We found that 32% of females and 23% of males reported 100% certainty of reaching age 85, and average expected survival chance exceeded the statistically predicted survival chance for 23% of males and 16% for females in age-ranges 50–60 and 61–70. Our multivariable analysis found that health literacy, internal health locus of control, willingness to take health risks, self-rated health, and health and life satisfaction all showed a significant positive association with expectation of reaching age 85. Moreover, those on daily medications, ex- or current smokers, and heavy drinkers were significantly less optimistic about reaching age 85.

**Conclusions:**

Particularly for the population groups with inaccurate life expectancies, the significant associations with psychological and behavioral factors open a way for initiatives based on behavior change theories to reach a better agreement between subjective and statistical life expectancy.

## Background

The United Nations Decade of Healthy Ageing (2021–2030) aims to improve the lives of older people, their families, and the communities in which they live through fostering healthy ageing, reducing inequity, changing unhealthy behaviors, and fostering consumption and savings behavior [1]. One of the key determinants of consumption and savings behavior, and perhaps also a person's own health and health behaviors, is subjective life expectancy (SLE), which is a measure of an individual’s expectation of remaining years of life [2–4].

SLE may affect decisions and priorities many years before reaching an advanced age [5]. Both in the sense that an individual may try to improve some less life-supporting variables (e.g., stop smoking), but also in the sense that an individual may fatalistically view their savings and pensions or opt out of preventive treatment initiatives. From a societal perspective, it could be argued that the best possible agreement between subjective and statistical life expectancy is worth striving for, since that would allow people to be more optimistic and make well-informed choices regarding lifestyle, savings, and pensions, and to achieve healthy and successful ageing [6].

During the last 50 years, the average life expectancy of Danes has increased by nearly 10 years, as in other European countries [7]. According to most recent estimates from Statistics Denmark, the average life expectancies from birth for women and men in Denmark are 83.4 years and 79.6 years, respectively [8]. At age 55, the average statistical life expectancy is generally about 30 more years, i.e., 85 years. Differences between SLE and actuarial life expectancy is relevant regarding how population groups plan to spend and support the remaining part of their lives financially as well as health- and activity-wise. The initial aim of the present study is therefore to estimate what proportion of Danes between age 50 and 70 misestimate their chances of reaching the average statistical life expectancy of about 85 years.

Several studies have identified important variations in how well SLEs are predicted, which could be due to differences in people’s socioeconomic characteristics as well as differences in health care contexts, and their physical environment [9–14]. People’s SLE estimates are based on their interpretations of their individual experiences, and considering various lifestyle factors, these estimates tend to be reasonably accurate when compared to actual mortality rates [15]. Griffin et al.’s (2013) biopsychosocial framework provides an extensive list of determinants for perceived life expectancy including biomedical factors (age, sex, age of parents, disease status, body mass index), socioeconomic factors (income and education), health behaviors (smoking status, alcohol consumption, physical activity, and diet), and psychosocial factors (optimism, psychological distress, and social connectedness) [3, 16]. Most of these individual factors are well-established; some are adjustable by personal efforts, some affect the expected average life expectancy, and others affect the perceived quality of life [5, 9, 13, 14, 16–18].

Among the factors included in Griffin’s biopsychosocial model, not all behavioral and psychological factors are well understood regarding systematic differences among sub-populations in the accuracy of these anticipated SLE ratings [3, 16]. More relevant from the perspective of behavioral change theories would be health literacy, internal health locus of control (IHLC), and willingness to take health risks (WTHR), as these factors may directly influence current health behaviors as well as medical decision making (e.g., compliance in taking preventative treatments and, cancer screening), and they have higher potential for modifiability.

IHLC, for instance, represents the degree of personal responsibility relative to health and health outcomes; IHLC and health literacy are key elements of self-rated health as well as general health and well-being [19–21]. Furthermore, people also differ in their propensity for taking health risks, such as whether to take a preventive medication that potentially has side effects, participate in cancer screening tests, or undergo surgery [22].

Past empirical evidence, however, does not permit a conclusive answer regarding whether psychological traits such as attitudes towards health and healthcare in general, IHLC, and WTHR may predict individual expectations of reaching average life expectancy. Therefore, in this study, it is initially examined on the individual level whether SLE is in line with the statistically calculated chance of reaching age 85. Then, factors associated with under or overestimation are explored with an aim to inform policies and allow people to reach a higher agreement between their individual expectations of reaching age 85 and their statistical chances, thereby allowing for better life decisions.

## Methods

### Sample and procedure

We used a cross-sectional survey design based on a representative sample of 15,072 Danish citizens aged 50–80 years who were randomly selected and contacted through the national digital mailbox (e-Boks.dk) in 2019 [21]. A web-based standardized questionnaire was developed in Danish and administered by Statistics Denmark to collect the data, and socio-demographic data was added from a national registry. The questionnaire was sent only to individuals aged 50–80, and up to two reminders were sent through the digital mailbox. In the net sample, 7,204 persons (48%) returned a completed questionnaire.

We excluded individuals aged 71–80 years (*n* = 1,759) from the analysis, as it is ideally required that the question to measure expectation to reach age 85 is asked of individuals 10 to 15 years younger than the target age [3, 15, 16]. Of the total 5,445 respondents aged 50–70 responding to the survey, 5,379 individuals answered the question on SLE (response rate = 99%).

According to the Danish Act on a Biomedical Research Ethics Committee System, the project was not a biomedical research project and did not need the ethics committee’s approval. Data included information that could potentially identify individuals, and the project was therefore registered at the University’s Research and Innovation Office. Data handling was in accordance with the General Data Protection Regulation (EU 2016/679).

### Study variables and measurement

#### Outcome variable

##### *Subjective expectation of reaching age 85*

We measured the outcome variable “expectation of reaching age 85” by the following question: “Think a little ahead of time. On a scale of 0 to 10, how likely do you think it is that you will experience your 85^th^ birthday”. Participants could answer on a scale from 0 (will not happen) to 10 (will certainly happen). These answers were translated into anticipated chances of reaching age 85 from 0%, 10%, 20%, 30%, and so on to 100%. Reporting SLE between 0 and 100% could be biased, however, since there are non-linearities in individual subjective beliefs [10]. A 10% point change in reported probabilities was seen as helpful because it implies a considerable change in beliefs at certain points in the probability distribution, providing a more accurate linear mapping of subjective likelihood beliefs.

#### Independent variables

##### *Health literacy*

Health literacy was measured using a shorter version of the health literacy scale which included one item for each 4 aspects of chronic disease management, as suggested by Poureslami et al. [23]. The items were: (a) finding information about diseases, (b) finding professional help when ill, (c) a good understanding when communicating with physicians, and (d) a good understanding of how to take medications [21, 23]. All items were presented with 4-point scales (from 1 = very easy to 4 = very difficult). Item responses were summed up for the analysis. Cronbach’s alpha for the 4-item HL scale was 0.83.

##### *Internal health locus of control (IHLC)*

We used a 6-item IHLC sub-scale from the original 18-item Multidimensional Health Locus of Control Scale (Form C) [24]. The items were: (a) If my health worsens, it is my own actions which determines how soon I feel better again; (b) I have control over my own health; (c) If my health deteriorates, it is my own responsibility; (d) My own behavior is the primary factor that influences my health; (e) By taking good care of myself, I can reduce the likelihood of becoming ill; and (f) if I have the correct behavior, I can stay healthy and well”. All the items were rated on a 6-point Likert scale (1 = strongly disagree to 6 = strongly agree) and all the item responses were summed up for the analysis. Cronbach’s alpha for the 6-item IHLC scale was 0.84.

##### *Willingness to take health risks (WTHR)*

Across the commonly used risk propensity scales, literature indicates that most items are related to general risk taking behavior (preference of avoiding risks, frequency of taking risks, attitude towards risks etc.) except one item which is related to health risks [22, 25, 26]. WTHR was therefore assessed by this single question: “How do you evaluate your willingness to take a risk related to your health situation?” Participants could answer on a scale from 0 (no risk willingness) to 10 (high risk willingness) [26].

#### Other potential explanatory and confounding variables

As potential confounding variables, we included socio-demographic, biomedical, and behavioral and psychological variables that have previously been reported to be associated with SLE [5, 9, 13, 14, 16–18]. These variables were: age, sex, highest education, income, smoking, body mass index (BMI), taking daily medications, alcohol consumption, life and health satisfaction, self-rated health, and health-related quality of life (HR-QoL).

Age was grouped into two categories, 50–60 and 61–70. Highest educational attainment was grouped into five categories: “elementary school’’, “high school”, “vocational education”, and “higher education”), and average annual income was grouped into three categories: < €33,334”, €33,334–€46,666, and > €46,667. To measure use of daily medications, respondents were asked whether they used daily prescription medications, other than vitamins, minerals, omega-3 fatty acids, herbal medicines, and other equivalent products (“yes”, “no”). Further, BMI was calculated based on height and weight indicated by the respondents.

Smoking was assessed with a single item: “Do you smoke?”, and the variable was coded with three levels: “never smoked” “ex-smoker”, and “current smoker”. Alcohol consumption was assessed with a single item: “How many units (equivalent to one glass of wine) of alcohol do you usually drink in a week?”, and answers were categorized into “non-drinker”, “1–7 units”, “7–14 units”, “14–21 units” and “ > 21 units”.

HRQoL was measured using the five-level EuroQol five-dimensional questionnaire (EQ-5D-5L), which is a generic preference-based measure comprising five dimensions, namely mobility, self-care, usual activities, pain/discomfort, and anxiety/depression. Each dimension in the EQ-5D-5L has five response levels: no problem, slight problems, moderate problems, severe problems, and unable/extreme problems [27]. All the item responses were summed up for the analysis.

Satisfaction with life was assessed with the single item: “How satisfied are you, all in all, with your life?” and satisfaction with health was assessed with a single item: “How satisfied are you with your health?” Both questions were presented with response scales from 0 (lowest level of satisfaction) to 10 (highest level of satisfaction). Self-rated overall health was assessed using the single question: “How would you rate your current state of health?” which was rated on a 5-point Likert scale (1 = nearly perfect to 5 = very poor) [28].

### Statistical analyses

We used data from Statistics Denmark (dst.dk) to calculate the average probability of survival to age 85 for men and women aged 50-60 and 61-70 in 2020. Given that the study participants were asked to indicate their expected survival to 85 years on a scale from 0 to 10 (percentage chance of scale 0 = 0%, scale 10 = 100%), we calculated average SLE probabilities for each age and sex group and compared this to the actuarial data from Statistics Denmark.

To describe the categorical and continuous characteristics of the study sample, descriptive statistics (percentages, means, and SDs), chi-squared tests and *t* tests were used. Further, we conducted bivariate Pearson correlation and multiple linear regression analyses to examine the relationship between subjective expectation of reaching age 85 and formative and psychological factors. Significance levels for testing individual variables were set at *p*-value < 0.05. Tolerance and variance of inflation factors (VIF) were calculated to assess collinearity in the multivariate model, with tolerance values of < 0.10 and VIF values of > 10 indicating possible collinearity. All analyses were performed using Stata 17.0 (StataCorp LP, College Station, TX).

## Results

Table 1 shows the general characteristics of our respondents (*n* = 5,379). Altogether, 52% of the respondents were aged 50–60, 48% were aged 61–70, and females (54%) outnumbered males (46%) in our study participants. One in six (17%) had completed a minimum education level, i.e., elementary school, and one in three (33%) had an annual income lower than €33,334. Most of them were not on daily medications (55%), were slightly overweight (mean BMI = 27, SD = 4.8), were non-smokers (57%) and consumed fewer than 7 units of alcohol on a weekly basis (74%).

**Table 1:** Descriptive statistics

**Participant characteristics**	**All** *n* = *5,379*	**Men** *n* = *2,501*	**Women** *n* = *2,878*	***p* value**^a^
**Subjective expectation of reaching age 85** (range 0–10; mean/SD)	6.9 (2.9)	6.6 (3.0)	7.2 (2.8)	**< 0.001**
**Socio-demographic/biomedical factors**				
Age ranges, n (%)				0.178
50–60	2,778 (51.6)	1,267 (50.7)	1,511 (52.5)	
61–70	2,601 (48.4)	1,234 (49.3)	1,367 (47.5)	
Highest educational attainment, n (%)				**< 0.001**
Elementary school	938 (17.4)	438 (17.5)	500 (17.4)	
High school	2,651 (49.3)	1,368 (54.7)	1,283 (44.6)	
Vocational education	1,232 (22.9)	400 (16.0)	832 (28.9)	
Higher education	558 (10.4)	295 (11.8)	263 (9.1)	
Average personal income per year, n (%)				
< €33,334	1,754 (32.6)	734 (29.4)	1,020 (35.4)	**< 0.001**
€33,334 – €46,666	1,674 (31.1)	800 (32.0)	874 (30.4)	
> €46,667	1,951 (36.3)	967 (38.6)	984 (34.2)	
Taking medication daily, n (%)				0.066
No	2,864 (53.9)	1,297 (52.6)	1,567 (55.1)	
Yes	2,445 (46.1)	1,169 (47.4)	1,276 (44.9)	
BMI (range 15–63), mean (SD)	26.6 (4.8)	27.3 (4.3)	26.1 (5.1)	**< 0.001**
**Behavioral factors**				
Smoking habit, n (%)				**0.008**
Never smoked	3,027 (56.9)	1,353 (54.8)	1,674 (58.8)	
Ex-smoker	1,419 (26.7)	703 (28.4)	716 (25.2)	
Current smoker	872 (16.4)	415 (16.8)	457 (16.0)	
Alcohol consumption, n (%)				**< 0.001**
Non-drinker	1,360 (25.6)	445 (18.0)	915 (32.1)	
1–7 units per week	2,549 (48.0)	1,125 (45.6)	1,424 (50.1)	
7–14 units per week	809 (15.2)	438 (17.7)	371 (13.0)	
14–21 units per week	367 (6.9)	268 (10.9)	99 (3.5)	
> 21 units per week	230 (4.3)	193 (7.8)	37 (1.3)	
**Health literacy** (range 4–16), mean (SD)	13.1 (2.1)	12.9 (2.1)	13.3 (2.1)	**< 0.001**
**Psychological factors**				
ILHC (range 6–36), mean (SD)	21.5 (6.6)	22.5 (6.4)	20.6 (6.7)	**< 0.001**
WTHR (range 0–10), mean (SD)	6.9 (2.9)	5.9 (2.3)	7.2 (2.8)	**< 0.001**
HRQoL (range 7–25), mean (SD)	22.6 (2.8)	22.8 (2.6)	22.4 (2.9)	**< 0.001**
Self-rated health (range 1–5), mean (SD)	3.5 (0.9)	3.5 (0.9)	3.4 (1.0)	**< 0.001**
Life satisfaction (range 0–10), mean (SD)	8.1 (1.8)	8.1 (1.7)	8.1 (1.8)	0.807
Health satisfaction (range 0–10), mean (SD)	7.1 (2.4)	7.2 (2.2)	7.0 (2.5)	**0.029**

*BMI* Body mass index, *IHLC* Internal health locus of control, *WTHR* Willingness to take health risks, *HRQoL* Health-related quality of life

^a^Performed chi-Square test for categorical variable and t test for continuous variable

The mean score for health literacy was 13.1 (SD = 2.1) out of a maximum possible score of 16, while the mean score for IHLC was 21.5 (SD = 6.6) out of the maximal score of 36. On average, HRQoL scored 22.6 (SD = 2.8) out of the total possible score of 25, while self-rated health scored 3.5 (SD = 2.8) out of a maximal score of 5. Out of a maximal score of 10, WTHR, health satisfaction, and life satisfaction had average scores of 6.9 (SD = 2.9), 7.1. (SD = 2.4) and 8.1 (SD = 1.8), respectively.

### Subjective expectation of reaching age 85

**Fig. 1:**
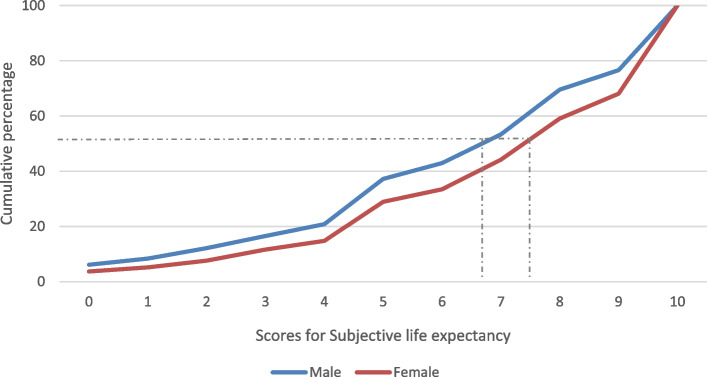
Cumulative percentage distribution of response scores to the question “chance of reaching age 85”

Across the entire sample (*n* = 5379), 28% of respondents reported a 100% certainty of living to age 85, while 5% reported a 0% chance of living to that age. Although the response distributions for males and females followed the same pattern, females exceeded males in their expectations, with 32% of women, as opposed to 23% of men, reporting 100% certainty of reaching age 85 (Fig. 1). Half of the male respondents viewed their chances of reaching age 85 as greater than 67%, whereas 50% of the females viewed their equivalent chances as greater than 75% (Fig. 1). The female curve is shifted to the right, indicating that females generally see their chances of reaching age 85 as higher than males. There was a significant difference in average scores for SLE between men and women (*p*-value < 0.05), with an average score of 6.9 for men and 7.2 for women (Table 1).

In both age ranges, the average self-reported chance of reaching age 85 exceeded the actuarial calculated average chance by 18% to 24% in males and 12% to 17% in females (Table 2). The range is the probability for the upper and lower age limit of the age range 50 to 70.

**Table 2:** Proportion of males and females of different age groups expected to reach age 85

Birth year	Age in 2020	Fraction living in 2020 expected to reach age 85^a^	Average self-reported chance of reaching age 85^b^
**Males**			
1961–70	50–60	42–44%	64%
1951–60	61–70	44–50%	68%
**Females**			
1961–70	50–60	56–57%	71%
1951–60	61–70	57–62%	74%

^a^Based on the period life table for Denmark 2020/2021 (Data Source: Statistics Denmark, www.dst.dk). The range indicates the probability for the lower and upper age limit of the group

^b^These figures are based on the data from the present study and pertain to the average of each respondent’s reported percentage of expectation of reaching age 85 across the age ranges 50–60 and 61–70

### Factors associated with subjective expectation of reaching age 85

The initial bivariate correlation analysis found that the psychological variables, namely HRQoL, health literacy, self-rated health, IHLC, increased satisfaction with life and health, and increased WTHR, were all significantly and positively associated with subjective expectation of reaching age 85 (Table 3). Significant positive correlations were also observed among other psychological variables.

**Table 3:** Bivariate correlation between health literacy, psychological variables, and subjective expectation of reaching age 85 (*n* = 5,379)

Variables	1	2	3	4	5	6	7	8
1. SLE	1.00							
2. WTHR	0.05**	1.00						
3. Life satisfaction	0.27**	0.28**	1.00					
4. Health satisfaction	0.46**	0.04**	0.50**	1.00				
5. Self-rated health	0.51**	0.06**	0.33**	0.64**	1.00			
6. HRQoL	0.45**	0.06**	0.33**	0.60**	0.72**	1.00		
7. Health literacy	0.19**	0.04**	0.17**	0.17**	0.21**	0.20**	1.00	
8. IHLC	0.23**	0.07**	0.14**	0.30**	0.32**	0.30**	0.09**	1.00

Variance inflation factor (VIF) < 2.5, for all variables

*SLE* Subjective life expectation, *WTHR* Willingness to take health risks, *HRQoL* Health related quality of life, *IHLC* Internal health locus of control

^**^p < 0.001

The subsequent multiple regression analysis (Table 4) between health literacy, psychological factors, and subjective expectation of reaching age 85 showed that health literacy, IHLC, and WTHR, as well as self-rated health and health and life satisfaction all had a significant and positive association (*p* < 0.05) with higher expectations of reaching age 85.

**Table 4:** Multiple regression analysis of heath literacy, psychological and behavioral factors, and subjective expectation of reaching 85 years (*n* = 5,379)

Variables	β	t value	95% CI	*p* value
Health literacy	0.07	4.18	0.04—0.10	< 0.001
ILHC	0.02	2.98	0.01—0.03	0.003
WTHR	0.05	3.15	0.02—0.07	0.002
Life satisfaction	0.23	9.23	0.18—0.28	< 0.001
Health satisfaction	0.37	13.47	0.32—0.42	< 0.001
Self-rated health	0.46	7.62	0.34—0.58	< 0.001
Sex, *female*	0.71	10.12	0.57—0.84	< 0.001
Smoking, e*x-smoker*	-0.22	-2.88	-0.37—-0.07	0.004
Smoking, c*urrent smoker*	-1.07	-11.33	-1.26—-0.89	< 0.001
Alcohol consumption, > *21 units per week*	-0.44	-2.54	-0.78—-0.10	0.011
Taking daily medications, *no*	0.26	3.60	0.12—0.40	< 0.001

Not significant variables: age, education, personal income, body mass index, and health-related quality of life

Model statistics, Prob > *F* = 0.0000; R-squared = 0.3869; *β* Regression coefficient, *CI* Confidence interval

*IHLC* Internal health locus of control, *WTHR* Willingness to take health risks

Additionally, behaviors such as use of daily medications, past or current smoking habits, and heavy drinking habits had a significant and negative effect on SLE. Females were positively associated with higher SLE, while no significant associations were found between SLE and age, education, income, BMI, or HR-QoL. We did not observe any significant multicollinearity problems in the multivariable model (VIF < 10).

## Discussion

Our findings revealed that men and women aged 50 to 70 generally overestimate their chance of reaching age 85 compared to their actuarial/statistical survival probability. Expected survival probability exceeded the statistical survival probability by 18% to 24% in males (aged 50–70) and 12% to 17% in females (aged 50–70). In agreement with population statistics, women generally rated their chances of reaching age 85 as higher than men (*p*-value < 0.05), and females (32%) outnumbered males (23%) in their estimation of 100% certainty of reaching age 85. Higher optimism regarding reaching age 85 is positively associated with psychological factors such as health literacy, IHLC, WTHR, self-rated health, and health and life satisfaction, and negatively associated with behaviors such as use of daily medications, past or current smoking habits, and heavy drinking habits.

It could be argued that men are generally aware of their lower life expectancy so that they also present lower confidence in reaching age 85 [29]. However, our study suggested that it is not just the biological sex difference in survival chances, but also the associated behaviors as well as psychological characteristics that are important.

While examining which population groups are more or less accurate in their SLE, we found that expected survival probability exceeded the statistical survival probability by 23% in males (aged 50–70) and 16% in females (aged 50–70), suggesting that a higher proportion of males are relatively less accurate and more prone to overestimating their survival chance than females. Consistent with previous European studies, individuals generally overestimate their likelihood of living to a given age, but men to a greater extent than women [5, 10, 15, 17]. The overall agreement with previous European studies suggests validity of the present study, despite the fact that there is heterogeneity between the European studies in terms of target age in the SLE question as well as different target age groups [5, 9, 10, 13, 14, 16–18].

Generally, men seem to misestimate their chances of reaching age 85 much more than women, however men, in our study sample, had relatively better income status than women. In fact, the observed difference in optimism between women and men, as found in our multivariable model, is not explained by higher income or even education, but rather by interrelated lifestyle factors such as daily medication use, smoking, and heavy drinking, which were disproportionately higher in men than in women (see Table 1). We could argue that our respondents more readily take into consideration behaviors such as daily medication use, smoking, and heavy drinking, but not their income and education, when estimating their life expectancy.

The behavioral characteristics and mechanisms that are unique to men and women of all ages and that relate to their survival probabilities have been discussed previously in a narrative review [29]. It is interesting that we also, in the present study, observe that respondents aged 50 to 70 apparently adjust their expectations to known lifestyle factors that reduce life expectancy, such as previous smoking history, drinking behavior, and daily medication use. More relevant to our target population is that for the population sub-groups who are less optimistic about living to age 85 and are engaged in non-supportive health behaviors, it may seem to make little sense to be committed to maintain health and save up for a long life in retirement [30].

However, an important caveat to any direct transferal of SLE data on the population level to an individual level is that we do not know (nor do the respondents themselves, obviously) who will reach age 85. A significant fraction of the less optimistic population may indeed live beyond age 85 but potentially be less prepared for a longer lifespan, both financially and health-wise. Therefore, current public health efforts to reduce harmful exposures and modify behaviors through public policy initiatives are important but may need to be supplemented with information on savings and pensions [31].

The present study also indicates that such public health communication programs need to address underlying relevant psychological factors and low health literacy levels, that is to say, psychosocial behavioral intervention [32]. In addition to previously known psychological factors such as self-rated health, HR-QoL, and health and life satisfaction [19–21], the current paper adds new information that health literacy, IHLC and WTHR help to estimate a realistic SLE. For instance, in our multivariable model, despite the fact that health literacy, IHLC and WTHR had a weaker effect on SLE compared to previously known factors such as self-rated health and health and life satisfaction, these factors are still highly relevant, particularly regarding maintaining healthy behaviors in our target population, as suggested by a previous Danish study by Nielsen et al. (2022) [33].

Maintaining healthy behaviors among older patients generally requires commitment on the part of patients to follow treatment protocols, engage in self-care, eat a healthy diet, engage in physical activity, and to bear the associated costs. As previously published literature from Denmark [21, 33] and elsewhere [34] indicate, individuals with increased health literacy, IHLC and WTHR are more compliant with prescribed behaviors and medical treatment regimens due to increased self-efficacy and expectation of living a long disease-free life. In this context, the overall findings of the present study can be utilized for policy interventions for healthy aging or the overall well-being of aging people.

### Strengths and limitations

The relatively large sample with a range of relevant psychological variables is an important strength of this study. Still, the results should be interpreted within the context of its limitations. First, people may have different perceptions and preferences regarding whether to choose between living longer in poorer health, or living a shorter life in better health (e.g., time trade-offs). In this regard, the questions posed may not fully reflect these perspectives. Furthermore, only a single target age i.e., age 85 was used. Given that there is significant life expectancy difference between men and women in Denmark, multiple target ages -as in some of the referred literature [13]- may have provided richer information.

Further, due to the cross-sectional design, conclusions about causality and temporality cannot be drawn. Also, the study was based on a questionnaire completed at home, so we also cannot exclude the possibility that some respondents received assistance in completing the questionnaire. Information on age as a continuous variable was not made available to us, which only allowed for grouping respondents into a 10 year age range in the final multivariable model. We performed a re-analysis of the model including individuals aged 71–80 to assess whether having the target age fewer than 10–15 years ahead might modify responses to the SLE question. This sensitivity analysis revealed that the findings were similar. We included several closely related psychological variables in the multivariable model, but multi-collinearity did not emerge in our model.

## Conclusion

Danish men and women aged 50–70 generally overestimate their chance of reaching age 85 compared to their actuarial/statistical survival probability, with women being closer to their statistical survival chance. In addition to well-known factors from the literature, health literacy and psychological factors, such as IHLC and WTHR, show statistical linear associations with subjective life expectancy. With a general aim to improve agreement between statistical and perceived life expectancies, the associations with psychological and behavioral factors indicate a potential to reduce inaccuracy about survival chance via public health efforts to increase health literacy or change lifestyle behaviors.

## Data Availability

The datasets used and/or analyzed during the current study are available from the corresponding author on reasonable request.

## Abbreviations

BMI: Body mass index
HRQoL: Health-related quality of life
IHLC: Internal health locus of control
SLE: Subjective life expectancy
VIF: Variance inflation factor
WTHR: Willingness to take health risks
